# c-Abl Inhibition Delays Motor Neuron Degeneration in the G93A Mouse, an Animal Model of Amyotrophic Lateral Sclerosis

**DOI:** 10.1371/journal.pone.0046185

**Published:** 2012-09-25

**Authors:** Ryu Katsumata, Shinsuke Ishigaki, Masahisa Katsuno, Kaori Kawai, Jun Sone, Zhe Huang, Hiroaki Adachi, Fumiaki Tanaka, Fumihiko Urano, Gen Sobue

**Affiliations:** 1 Department of Neurology, Nagoya University Graduate School of Medicine, Tsurumai-cho, Showa-ku, Nagoya, Japan; 2 Program in Gene Function and Expression, University of Massachusetts Medical School, Worcester, Massachusetts, United States of America; 3 Core Research for Evolutional Science and Technology, Japan Science and Technology Agency, Saitama, Japan; 4 Program in Molecular Medicine, University of Massachusetts Medical School, Worcester, Massachusetts, United States of America; University of Edinburgh, United Kingdom

## Abstract

**Background:**

Amyotrophic lateral sclerosis (ALS) is a fatal neurodegenerative disease characterized by progressive death of motor neurons. Although the pathogenesis of ALS remains unclear, several cellular processes are known to be involved, including apoptosis. A previous study revealed the apoptosis-related gene c-Abl to be upregulated in sporadic ALS motor neurons.

**Methodology/Findings:**

We investigated the possibility that c-Abl activation is involved in the progression of ALS and that c-Abl inhibition is potentially a therapeutic strategy for ALS. Using a mouse motor neuron cell line, we found that mutation of Cu/Zn-superoxide dismutase-1 (SOD1), which is one of the causative genes of familial ALS, induced the upregulation of c-Abl and decreased cell viability, and that the c-Abl inhibitor dasatinib inhibited cytotoxicity. Activation of c-Abl with a concomitant increase in activated caspase-3 was observed in the lumbar spine of G93A-SOD1 transgenic mice (G93A mice), a widely used model of ALS. The survival of G93A mice was improved by oral administration of dasatinib, which also decreased c-Abl phosphorylation, inactivated caspase-3, and improved the innervation status of neuromuscular junctions. In addition, c-Abl expression in postmortem spinal cord tissues from sporadic ALS patients was increased by 3-fold compared with non-ALS patients.

**Conclusions/Significance:**

The present results suggest that c-Abl is a potential therapeutic target for ALS and that the c-Abl inhibitor dasatinib has neuroprotective properties *in vitro* and *in vivo*.

## Introduction

Amyotrophic lateral sclerosis (ALS) is a neurodegenerative disease characterized by selective loss of upper and lower motor neurons in the cerebral cortex, brain stem, and spinal cord [1], [2]. Many genes have been identified as involved in familial ALS cases, including Cu/Zn-superoxide dismutase-1 (SOD1) [3], [4], [5]. Approximately 5–10% of ALS cases are familial, and 20% of familial ALS cases are associated with mutations in the SOD1 gene [3]. Several hypotheses for the pathogenesis of ALS have been proposed, including oxidative stress, glutamate excitotoxicity, mitochondrial dysfunction, and neuroinflammation, all of which eventually lead to the death of motor neurons [6], [7], [8], [9]. Many studies using mutant SOD1 transgenic animals have explored the precise cellular mechanisms of motor neuron death; however, no therapeutic drugs have been developed to date except for riluzole, which has only limited effects. Since most cases of ALS are sporadic, the development of ALS drug therapies based on the pathology of sporadic ALS (sALS) is feasible.

Previously, we performed microarray analyses combined with laser-capture microdissection to investigate the gene expression profiles of spinal motor neurons isolated from autopsied patients with sALS [10]. We found altered expression of many genes, including dynactin 1, early growth response-3, acetyl-CoA transporter, death receptor 5, and cyclin C [10], [11]. In that study, a 4.41-fold increase in the amount of c-Abl mRNA was detected in the motor neurons of sALS patients [10]. These findings raised the possibility that upregulation of c-Abl in motor neurons contributes to motor neuron degeneration and that activation of this pathway may be one of the pathologic features of ALS.

c-Abl is a ubiquitous non-receptor tyrosine kinase that was originally identified as the cellular homolog of the v-abl gene, an oncogene carried by the Abelson murine leukemia virus [12]. Bcr-Abl hybrid protein, which is one of the oncogenic forms of c-Abl fusion kinase, causes chronic myelogenous leukemia (CML) and Philadelphia chromosome-positive adult acute lymphoblastic leukemia (Ph+ALL) [13], [14]. The kinase activity of c-Abl is regulated by phosphorylation. Tyrosine 245 (Tyr245) and tyrosine 412 (Tyr412) are well-established regulatory phospho-tyrosine residues that are required for c-Abl activation [15]. In response to various stimuli, c-Abl regulates cytoskeletal rearrangement, cell migration, cell-cell adhesion, cell proliferation, and apoptosis [16], [17], [18], [19], [20]. On exposure to stressors, such as DNA damage or oxidative stress, c-Abl has been implicated in cell growth arrest and caused apoptotic cell death in association with p73 [21], [22], PKC delta [23], and CDK5 [24], [25]. Recently, neural functions of c-Abl have also been described: c-Abl participates in neuronal development and neurite outgrowth [26], [27], and has also been implicated in the pathogenesis of Alzheimer's disease [28], [29].

In the present study, we investigated c-Abl activation in a mutant SOD1 transgenic ALS mouse model and in sALS patients, and we demonstrated that the c-Abl inhibitor dasatinib has a protective effect on motor neuron degeneration in G93A-SOD1 transgenic ALS mice (G93A mice).

## Results

### Inducible expression of wild-type and mutant SOD1 in NSC-34 cells

**Figure 1:**
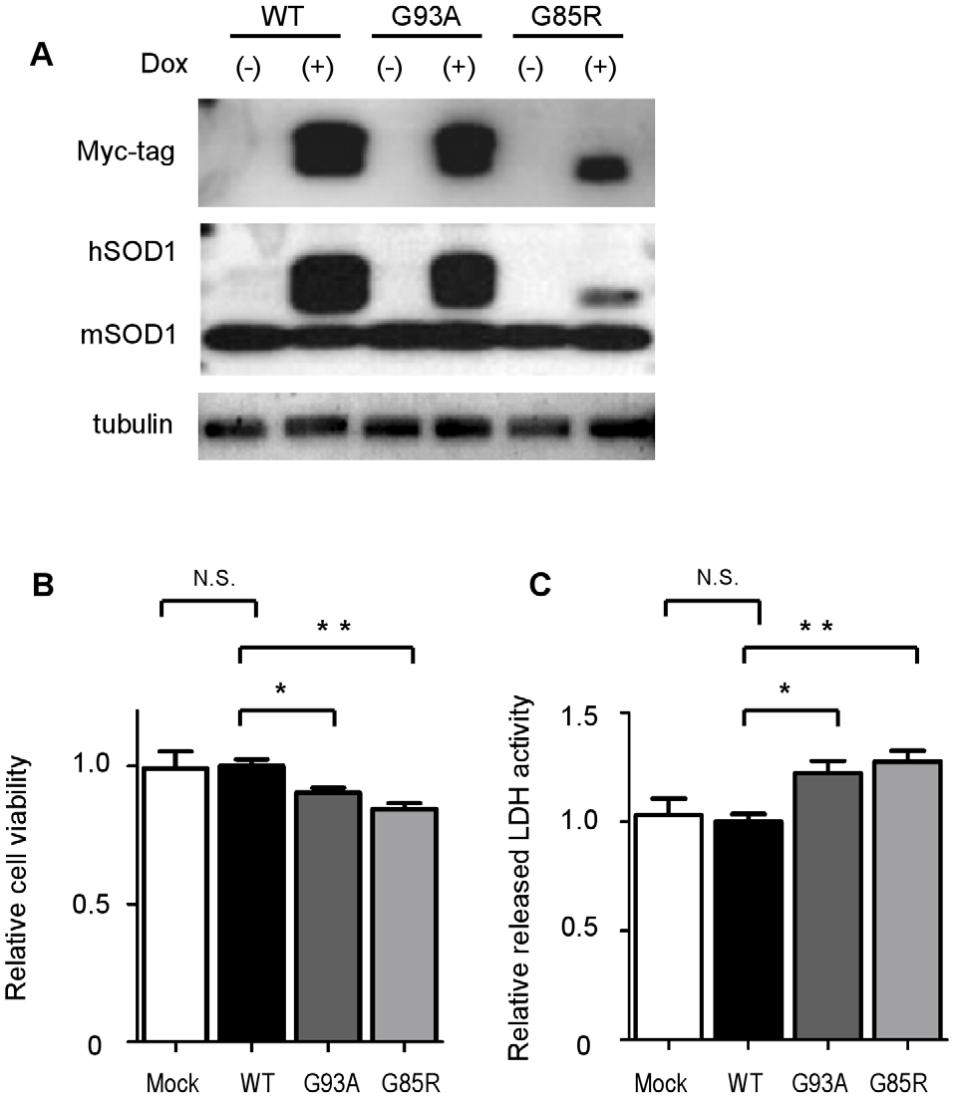
Inducible expression system of wild-type and mutant SOD1s in NSC-34 cells. A: NSC-34 cells were stably transduced with an inducible lentivirus expressing human Myc-tagged wild-type or mutant SOD1 protein. Cells were cultured with or without doxycycline (Dox, 2 µg/ml) for 48 h to induce SOD1 protein. Tubulin is shown as a loading control. hSOD1 and mSOD1 indicate human SOD1 and mouse endogenous SOD1, respectively. B: Cell viability assay based on the MTS method showed that overexpression of both types of mutant SOD1, G93A and G85R, caused cytotoxicity in serum-free culture medium. Mock indicates mock-transfected NSC-34 cells. Data are presented as mean ± SEM. Statistics were evaluated using 1-way ANOVA with Dunnett's post-hoc test. *P<0.05, **P<0.01 C: Cytotoxicity detection assay using the LDH release method revealed that overexpression of both types of mutant SOD1, G93A and G85R, caused cytotoxicity in serum-free culture medium. Data are presented as mean ± SEM. Statistics were evaluated using 1-way ANOVA with Dunnett's post-hoc test. *P<0.05, **P<0.01.

To investigate the expression and activity levels of c-Abl in human mutant SOD1-expressing motor neurons, we established an inducible system of NSC-34 cells able to express either human wild-type or mutant (G93A, G85R) SOD1 protein. Western blot analysis confirmed that myc-tagged human SOD1 proteins were induced by doxycycline in these cell lines (Fig. 1A). Myc-tagged human SOD1 demonstrated lower mobility than mouse endogenous SOD1. NSC-34 cells were well differentiated in low-serum medium with extended neuritic processes, a morphological marker of neuronal cell maturation and differentiation [30]. As a motor neuron-mimicking model, we used NSC-34 cells with serum-free medium to measure cytotoxicity. Cell viability was examined using the MTS-based cell proliferation assay at 48 h after the induction of SOD1 proteins, and we found that both G93A and G85R mutant SOD1s significantly decreased cell viability in comparison with wild-type SOD1 (*P*<0.05 for G93A, *P*<0.01 for G85R) (Fig. 1B). The cytotoxicity of mutant SOD1s was also measured by lactate dehydrogenase (LDH) release assay at 48 h after the induction of SOD1 proteins. The results demonstrated that both G93A and G85R mutant SOD1s significantly increased cytotoxicity in comparison with wild-type SOD1 (*P*<0.05 for G93A, *P*<0.01 for G85R) (Fig. 1C).

### c-Abl activation caused by mutant SOD1 in NSC-34 cells

**Figure 2:**
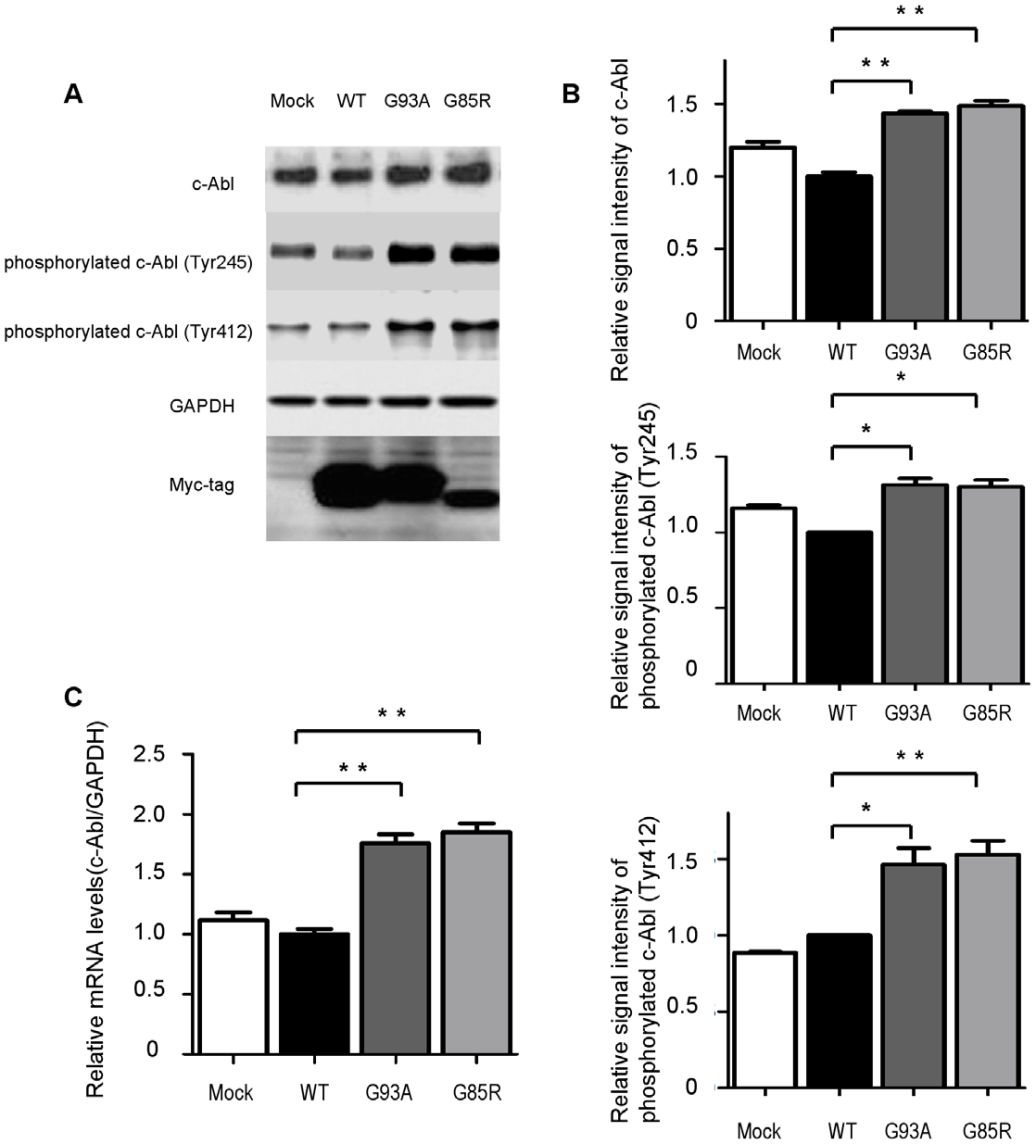
Activation of c-Abl caused by mutant SOD1 overexpression. A: Total c-Abl and phospho-c-Abl (Tyr245 and Tyr412) protein levels in NSC-34 cells overexpressing human wild-type and mutant SOD1 protein were measured by western blotting. GAPDH is shown as a loading control. Cells were cultured with doxycycline (Dox, 2 µg/ml) in serum-free culture medium for 48 h. B: Densitometric analysis (n = 3 per group) of the results shown in Fig. 2A demonstrated that both types of mutant SOD1, G93A and G85R, significantly increased the amount of total c-Abl protein and facilitated phosphorylation at both c-Abl sites, Tyr245 and Tyr412. Data are presented as mean ± SEM. Statistical analysis was performed using 1-way ANOVA with Dunnett's post-hoc test. *P<0.05, **P<0.01. C: Expression levels of c-Abl mRNA were measured by quantitative RT-PCR in NSC-34 cells overexpressing wild-type or mutant human SOD1 (n = 4 per group). Cells were cultured with doxycycline (Dox, 2 µg/ml) in serum-free culture medium for 48 h. Overexpression of both types of mutant SOD1 significantly increased the c-Abl mRNA level compared with overexpression of wild-type SOD1 (*P*<0.01). Data shown are ratios (mean ± SEM) of the c-Abl mRNA levels in NSC-34 cells overexpressing wild type SOD1 (n = 6). Statistics were evaluated using 1-way ANOVA with Dunnett's post-hoc test. **P<0.01.

We then investigated whether overexpression of mutant SOD1s influenced the expression of c-Abl. Western blot analysis revealed that the expression of c-Abl was greater in cells expressing mutant SOD1s (G93A and G85R) than cells expressing wild-type SOD1 (Fig. 2A). These differences were much more prominent when phospho-specific antibodies for each of 2 distinct tyrosine residues (Tyr245 and Tyr412) were used for the western blot analysis. Densitometric analysis confirmed that mutant SOD1 significantly increased the expression and phosphorylation of c-Abl (*P*<0.05) (Fig. 2B). Increased c-Abl mRNA expression in cells overexpressing mutant SOD1s was also confirmed by quantitative RT-PCR (Fig. 2C).

### Dasatinib attenuates the cytotoxicity of mutant SOD1s in NSC-34 cells

**Figure 3:**
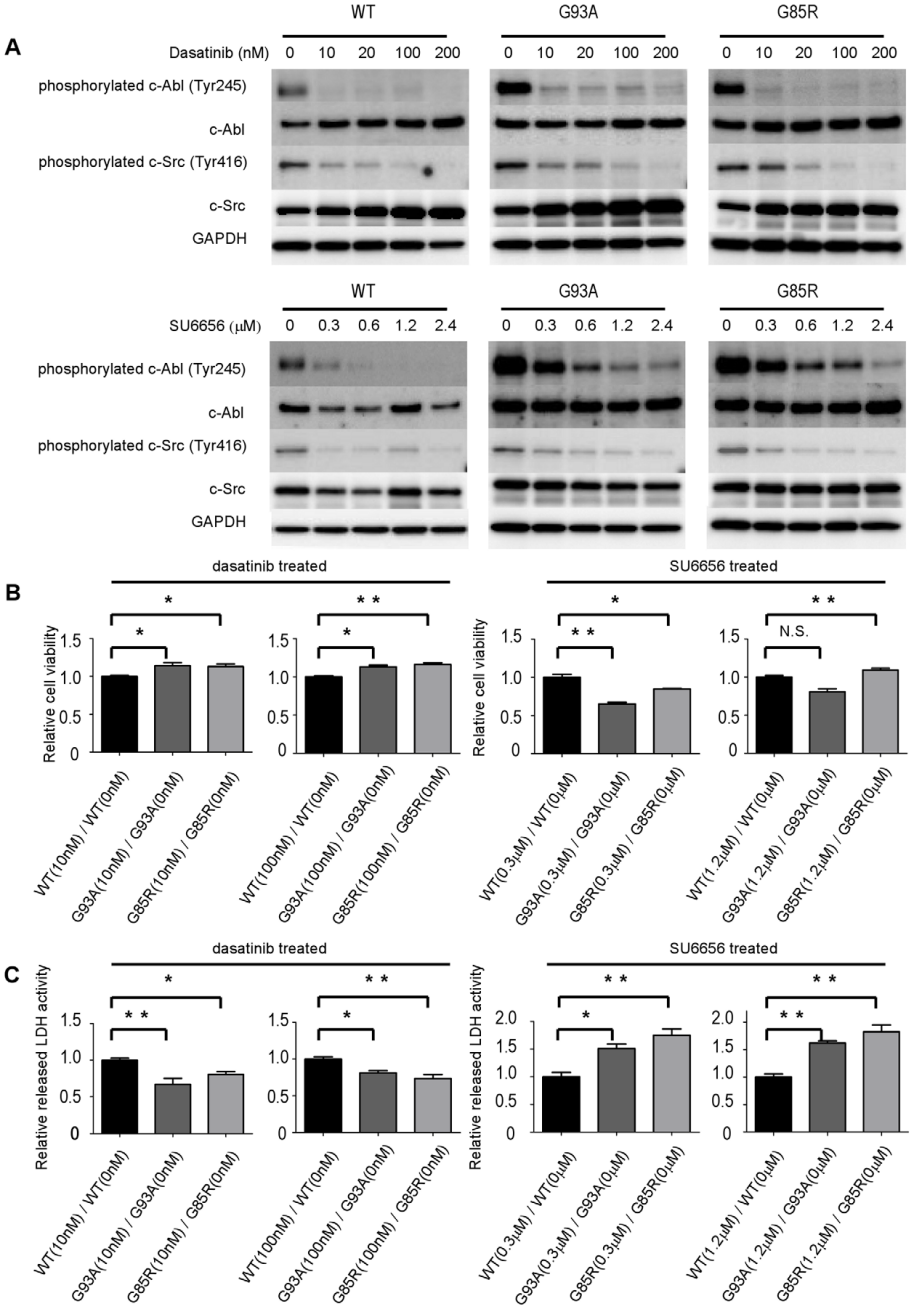
Dasatinib reduces cytotoxicity of mutant SOD1s in NSC-34 cells. A: Protein levels of phosphorylated c-Abl (Tyr245), c-Abl, phosphorylated c-Src (Tyr416), c-Src, and GAPDH in NSC-34 cells overexpressing human wild-type or mutant SOD1s treated with various concentrations of dasatinib or SU6656 were measured by western blot. Cells were cultured in serum-free culture medium with doxycycline (Dox, 2 µg/ml), and western blot was performed at 24 h after dasatinib or SU6656 addition. B: Cells were grown in 96-well collagen-coated plates (3,500 cells per well) with doxycycline (Dox, 2 µg/ml) in culture medium containing 10% FBS for 16 h. Culture medium was then replaced with 1% FBS-containing medium including the indicated concentrations of dasatinib and 2 µg/ml doxycycline (Dox). MTS assays were performed at 24 h after addition of dasatinib or SU6656. Viability was measured as the level of absorbance at 490 nm. Absorbance at 490 nm was expressed as the mean ± SEM (n = 6). Ratios of relative cell viability based on the MTS assay were calculated to determine the beneficial effect of dasatinib in mutant cells overexpressing SOD1s. Absorbance at 490 nm was standardized relative to the absorbance at each corresponding time point for 0 nM dasatinib. Cell viability assay confirmed that dasatinib significantly reduced the cytotoxicity of mutant SOD1s, whereas SU6656 did not. Statistics were evaluated using 1-way ANOVA with Dunnett's post-hoc test. *P<0.05, **P<0.01. C: Cells were grown in 96-well collagen-coated plates (3,500 cells per well) with doxycycline (Dox, 2 µg/ml) in culture medium containing 10% FBS for 16 h. Culture medium was then replaced with 1% FBS-containing medium with the indicated concentrations of dasatinib and 2 µg/ml doxycycline (Dox). LDH assays were performed at 24 h after dasatinib or SU6656 addition. Cytotoxicity was measured as the level of absorbance at 490 nm. Ratios of relative LDH release were calculated to determine the beneficial effect of dasatinib in mutant cells overexpressing SOD1s. Absorbance at 490 nm was standardized relative to the absorbance at each corresponding time point for 0 nM dasatinib. LDH assay confirmed that dasatinib significantly reduced the cytotoxicity of mutant SOD1s, whereas SU6656 did not. Values represent the mean ± SEM of the ratio of LDH release (n = 4). Statistics were evaluated using 1-way ANOVA with Dunnett's post-hoc test. *P<0.05, **P<0.01.

To examine whether the inhibition of c-Abl kinase influenced the cytotoxicity of mutant SOD1s, we evaluated the effect of dasatinib, a blood-brain barrier (BBB)-permeable c-Abl inhibitor, on c-Abl activity in NSC-34 cells expressing different forms of SOD1. Cells overexpressing SOD1 were treated with increasing concentrations of dasatinib for 24 h and analyzed by western blotting. Dasatinib effectively suppressed the phosphorylation of c-Abl in all cell lines (Fig. 3A). Since dasatinib is a dual c-Abl/c-Src kinase inhibitor [31], in order to clarify the specificity of c-Abl for motor neuronal cytotoxicity, we also performed cell proliferation and cell death assays with SU6656, which preferentially inhibits c-Src compared to c-Abl. SU5666 effectively suppressed the phosphorylation of c-Src in all cell lines (Fig. 3A). Cell viability and cell death assays confirmed that dasatinib significantly reduced the cytotoxicity of mutant SOD1s (*P*<0.05), whereas SU6656 did not (Fig. 3B, C).

### Upregulation and activation of c-Abl in G93A mice

**Figure 4:**
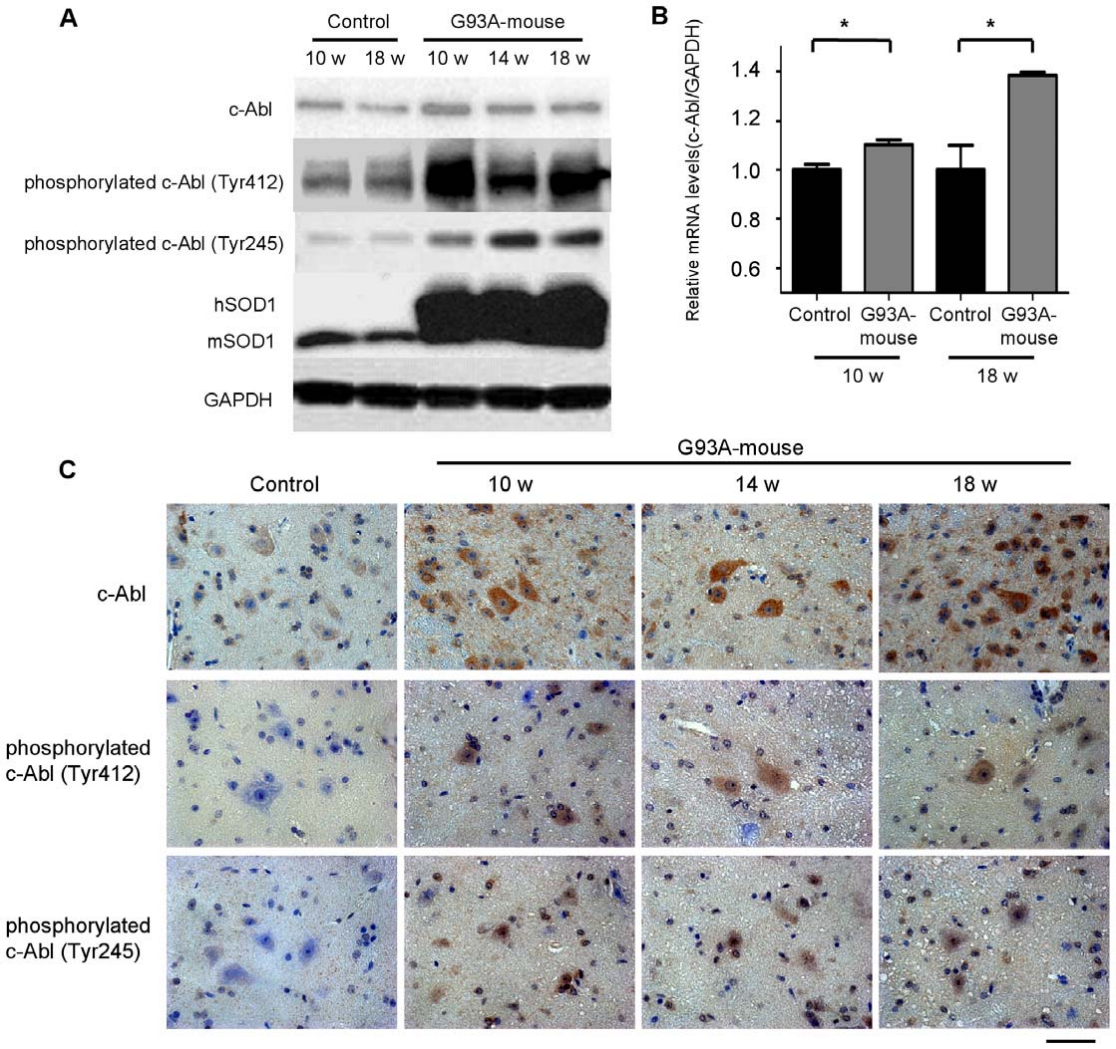
c-Abl upregulation and activation in G93A mice. A: Protein levels of phosphorylated c-Abl (Tyr245 and Tyr412) and c-Abl were analyzed by western blot using spinal cord protein extracts from control non-transgenic and G93A mice at the indicated ages. GAPDH is shown as a loading control. hSOD1 and mSOD1 indicate human SOD1 and mouse endogenous SOD1, respectively. B: c-Abl mRNA levels in the spinal cords of G93A mice and control littermates (age 10 and 18 weeks; n = 4 per group) were measured by quantitative RT-PCR. Data shown are the ratios of the c-Abl mRNA level in each group relative to that in control littermates. c-Abl mRNA was significantly increased in the spinal cords of G93A mice in both age groups compared with control littermates (*P*<0.05). Data are presented as mean ± SEM. Statistics were evaluated using Student's *t* test. *P<0.05. C: Distribution of total and phosphorylated c-Abl proteins was analyzed by immunohistochemical staining of paraffin-embedded spinal cord sections from G93A mice (10, 14, and 18 weeks old) and control littermates (20 weeks old) using antibodies directed against c-Abl, phosphorylated c-Abl (Tyr245), and phosphorylated c-Abl (Tyr412). Scale bar: 50 µm.

To determine whether c-Abl upregulation also occurs in G93A mice, we measured mRNA and protein levels of c-Abl in the lumbar spinal cords of G93A and control mice at age 10 weeks (pre-symptomatic stage), 14 weeks (symptomatic stage), and 18 weeks (terminal stage) by quantitative RT-PCR and western blot analyses. The protein expression of c-Abl in the lumbar spinal cords of G93A mice was increased as early as 10 weeks compared with control littermates (Fig. 4A). A remarkable increase in the phosphorylation of c-Abl was also evident even at the pre-clinical stage of 10 weeks. The increase in c-Abl protein was paralleled by an induction of c-Abl mRNA in the spinal cords of G93A mice (Fig. 4B). Consistent with the western blot analyses and quantitative RT-PCR, immunoreactivity for c-Abl and phosphorylated c-Abl (Tyr245 and Tyr412) was increased in the lumbar spinal neurons of G93A mice compared with those of control littermates (Fig. 4C). We quantified the signal intensity of phosphorylated c-Abl immunofluorescence in motor neurons (Tyr412 and Tyr245) using Image J software. Phosphorylated c-Abl immunoreactivity in G93A mice was significantly increased compared to control mice with both antibodies (*P*<0.01), which indicated that c-Abl was activated at an early stage of disease in this mouse model of ALS (Fig. S1).

### The effect of dasatinib on survival and disease progression in G93A mice

**Figure 5:**
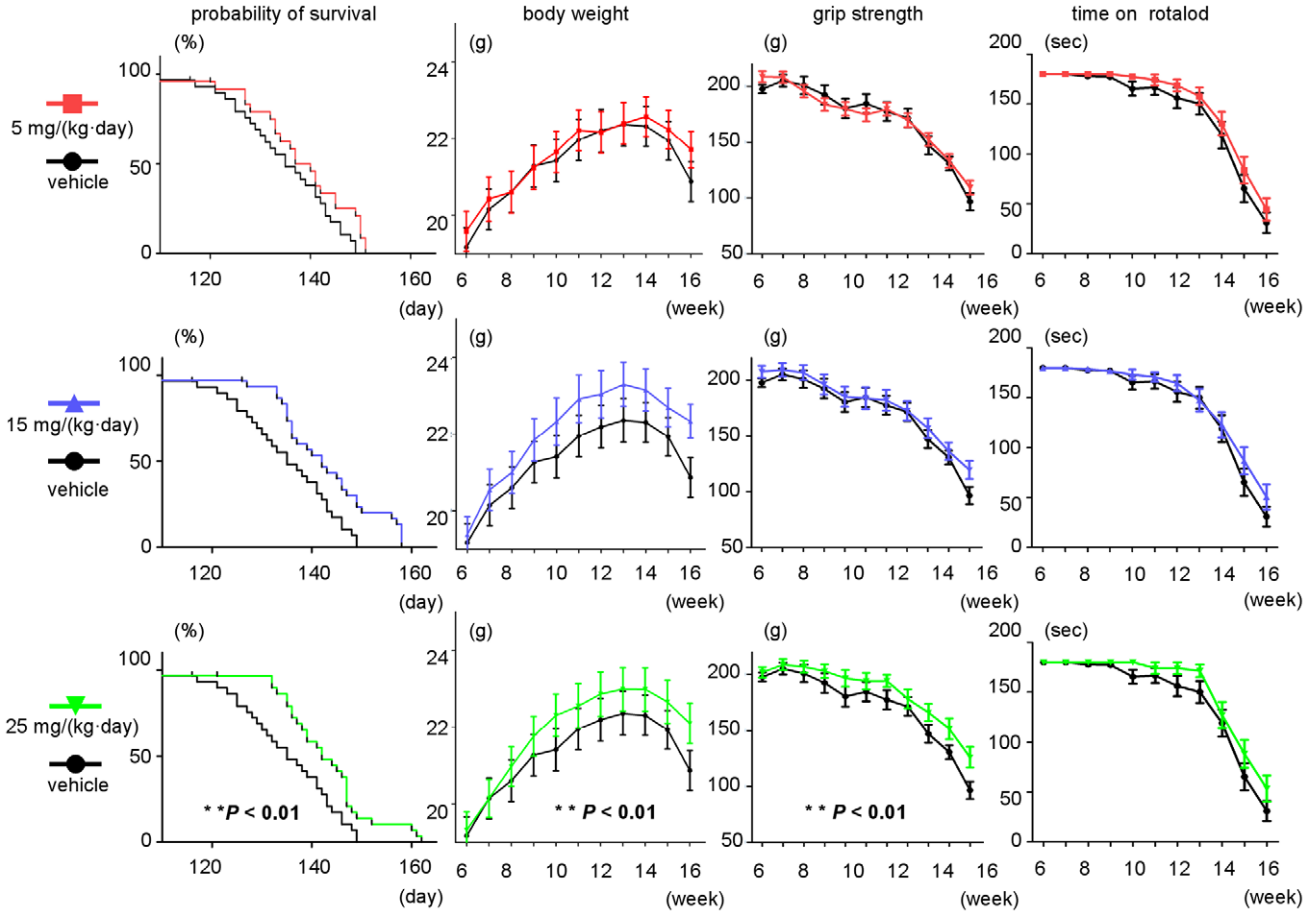
The effect of dasatinib on survival and disease progression in G93A mice. Rotarod activity, grip strength, body weight, and survival rate in G93A mice with or without dasatinib treatment (0, 5, 15, and 25 mg/(kg·day)). Survival of G93A mice was improved by dasatinib at a dose of 25 mg/(kg·day) compared with vehicle treatment (Log-rank test, *P*<0.01, 25 mg/(kg·day) vs. vehicle), whereas a lower dose of dasatinib (5 mg/(kg·day)) had no significant effect on life span. Weight loss was also ameliorated by dasatinib at a dose of 25 mg/(kg·day) compared with vehicle treatment (2-way ANOVA, *P*<0.01, 25 mg/(kg·day) vs. vehicle). The administration of dasatinib at 25 mg/(kg·day) similarly ameliorated grip strength (2-way ANOVA, *P*<0.01, 25 mg/(kg·day) vs. vehicle). The difference in physical function between the groups as assessed by rotarod was not significant by 2-way ANOVA, although a beneficial tendency of dasatinib was observed.

Survival of G93A mice was improved by dasatinib at a dose of 25 mg/(kg·day) compared with vehicle treatment (*P*<0.01, 25 mg/(kg·day) vs. vehicle), whereas a lower dose of dasatinib (5 mg/(kg·day)) had no significant effect on life span (Fig. 5). Weight loss was also ameliorated by dasatinib at a dose of 25 mg/(kg·day) compared with vehicle treatment (Fig. 5, 2-way ANOVA, *P*<0.01, 25 mg/(kg·day) vs. vehicle). The administration of dasatinib at 25 mg/(kg·day) similarly alleviated motor dysfunction measured by grip strength (2-way ANOVA, *P*<0.01, 25 mg/(kg·day) vs. vehicle). Dasatinib did not significantly ameliorate the physical function assessed by rotarod, although a beneficial tendency was observed. Dasatinib did not alter the neuromuscular function or body weight of non-transgenic littermates at any of the doses tested (data not shown).

### The effect of dasatinib on motor neuron survival and innervation status of neuromuscular junctions (NMJs) in G93A mice

**Figure 6:**
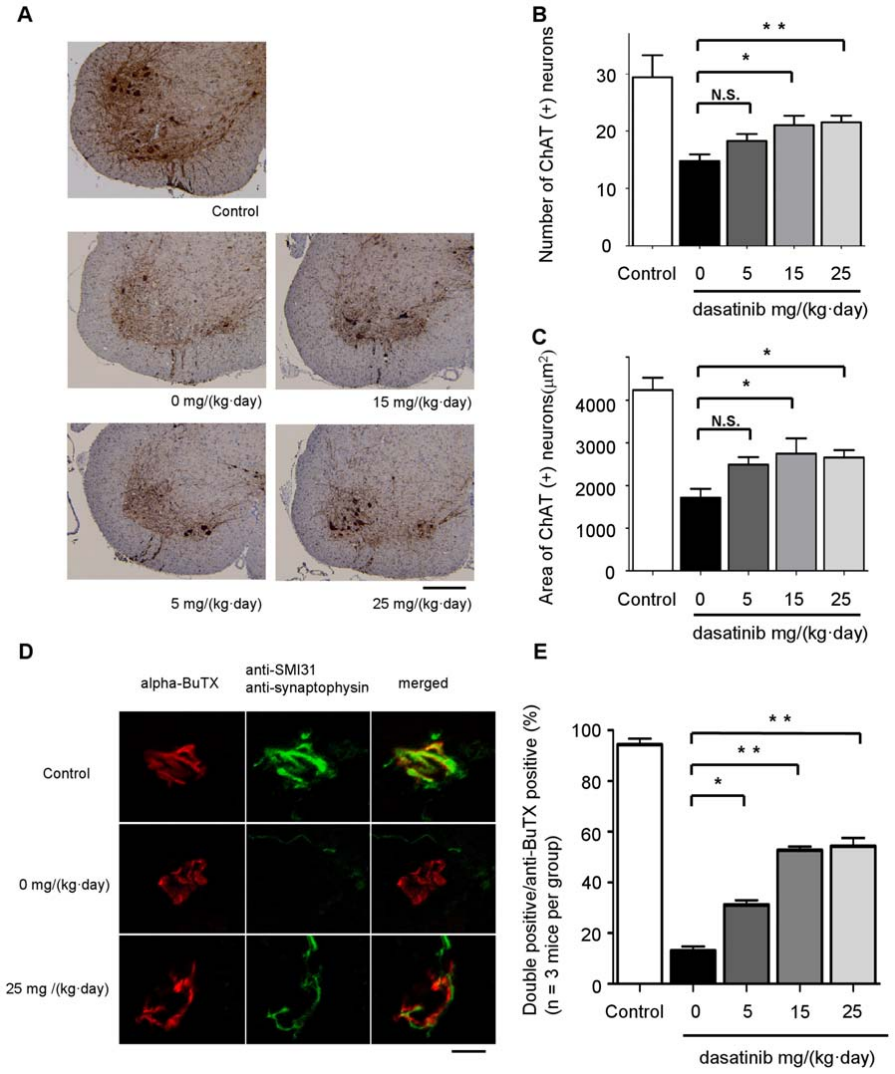
The effect of dasatinib on motor neuron survival in G93A mice. A: Spinal cord (L1-3) specimens from 120-day-old mice were immunostained with anti-ChAT antibody. The mice were administered the indicated amounts of dasatinib daily from postnatal day 56 to day 120 (n = 8 mice per group). Scale bar: 250 µm. B: The number of ChAT-positive neurons in the sections described in Fig. 6A was counted using Image J software. Dasatinib prevented the loss of ChAT-positive motor neurons in the ventral horn of G93A mice at doses of 15 mg/(kg·day) (*P*<0.05) and 25 mg/(kg·day) (*P*<0.01). Statistics were evaluated using 1-way ANOVA with Dunnett's post-hoc test. *P<0.05, **P<0.01. C: The area of ChAT-positive neurons in the sections described in Fig. 6A was determined using Image J software. Dasatinib increased the size of motor neuron cell bodies at doses of 15 and 25 mg/(kg·day) (*P*<0.05). Statistics were evaluated using 1-way ANOVA with Dunnett's post-hoc test. *P<0.05. D: To investigate the innervation status of NMJs, frozen quadriceps femoris specimens from 120-day-old mice were stained with alpha-BuTX (red) and anti-synaptophysin (green) or anti-SMI31 (green) antibodies. Representative NMJs visualized with the confocal laser scanning microscopy are shown. The mice were administered the indicated amounts of dasatinib daily from postnatal day 56 to day 120. Scale bar: 10 µm. E: The ratio of double-immunostained innervated NMJs to total NMJs is summarized. One hundred immunostained NMJs were investigated in each dasatinib-treated mouse (n = 3 mice per group). Dasatinib significantly ameliorated the destruction of NMJ innervation in G93A mice at doses of 5 (*P*<0.05), 15, and 25 mg/(kg·day) (*P*<0.01). Statistics were evaluated using 1-way ANOVA with Dunnett's post-hoc test. *P<0.05, **P<0.01.

Paraffin-embedded sections of the lumbar spinal cord (L1-3) from 120-day-old mice were analyzed immunohistochemically using anti-choline acetyltransferase (ChAT) antibody (Fig. 6A). The number of ChAT-positive motor neurons in the lumbar spinal cord was significantly preserved in mice treated with dasatinib at doses of 15 mg/(kg·day) or higher compared with vehicle-treated control mice (*P*<0.05) (Fig. 6B). To evaluate changes in the size of ChAT-positive motor neurons, we quantified the cell body areas of ChAT-positive motor neurons using Image J software. The size of motor neurons in dasatinib-treated mice was significantly preserved compared to vehicle-treated controls (*P*<0.05) (Fig. 6C). To investigate the innervation status of neuromuscular junctions (NMJs), frozen quadriceps femoris specimens were collected from 120-day-old mice and stained with alpha-bungarotoxin (BuTX) (red) and anti-synaptophysin (green) or anti-SMI31 (green) antibodies (Fig. 6D). We observed BuTX-positive NMJs (treated and control groups; n = 3 mice per group, 100 NMJs per mouse) using confocal laser scanning microscopy and counted double- (red and green) or single (red)-immunostained NMJs. Figure 6E summarizes the ratio of double-immunostained (innervated) NMJs to total NMJs. Dasatinib significantly ameliorated the destruction of NMJ innervation in G93A mice at doses of 5, 15, and 25 mg/(kg·day) compared to vehicle treatment (*P*<0.05) (Fig. 6E).

### Dasatinib reduces phosphorylation of c-Abl and the activated form of caspase-3 in G93A mice

**Figure 7:**
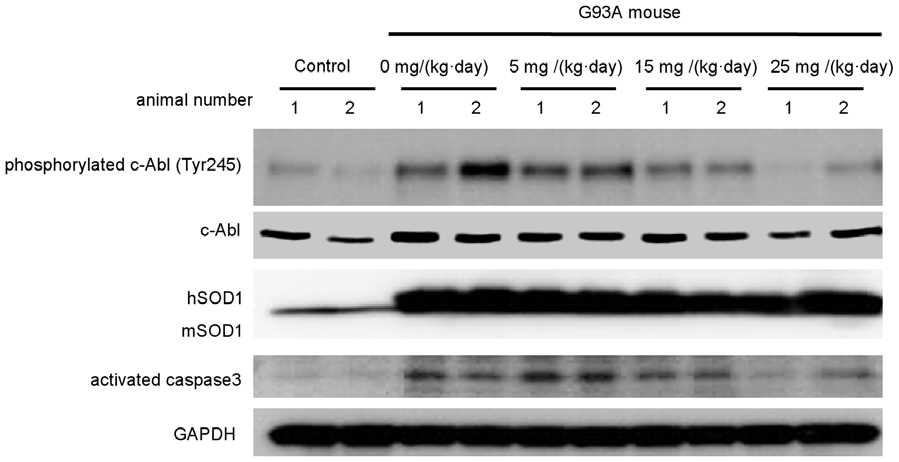
Dasatinib inhibits c-Abl phosphorylation in G93A mice. Protein levels of phosphorylated c-Abl (Tyr245), c-Abl, and activated caspase-3 were measured by western blot analysis using spinal cords from dasatinib- and vehicle-treated G93A mice (120 days old). GAPDH is shown as the loading control. hSOD1 and mSOD1 indicate human SOD1 and mouse endogenous SOD1, respectively. Western blot analysis is shown in duplicate. The animal number refers to individual animals. Parallel declines in c-Abl phosphorylation and activated caspase 3 were observed.

To assess the effect of dasatinib on the central nervous system (CNS), we performed western blot analyses using the spinal cords of G93A mice and control littermates treated with dasatinib or vehicle (Fig. 7). The levels of phosphorylated c-Abl (Tyr245) were decreased in a dose-dependent manner in G93A mice treated with dasatinib. In addition, activated caspase-3 was decreased in mice treated with high-dose dasatinib (Fig. 7). Quantification of immunofluorescence revealed that phosphorylated c-Abl (Tyr412) levels were significantly decreased in dasatinib-treated G93A mice at doses of 15 mg/(kg·day) or higher compared with vehicle-treated control mice (*P*<0.01) (Fig. S2). These results suggest that dasatinib protects motor neurons from mutant SOD1-induced neuronal cell death by inhibiting apoptosis.

### Upregulation and activation of c-Abl in sporadic ALS

**Figure 8:**
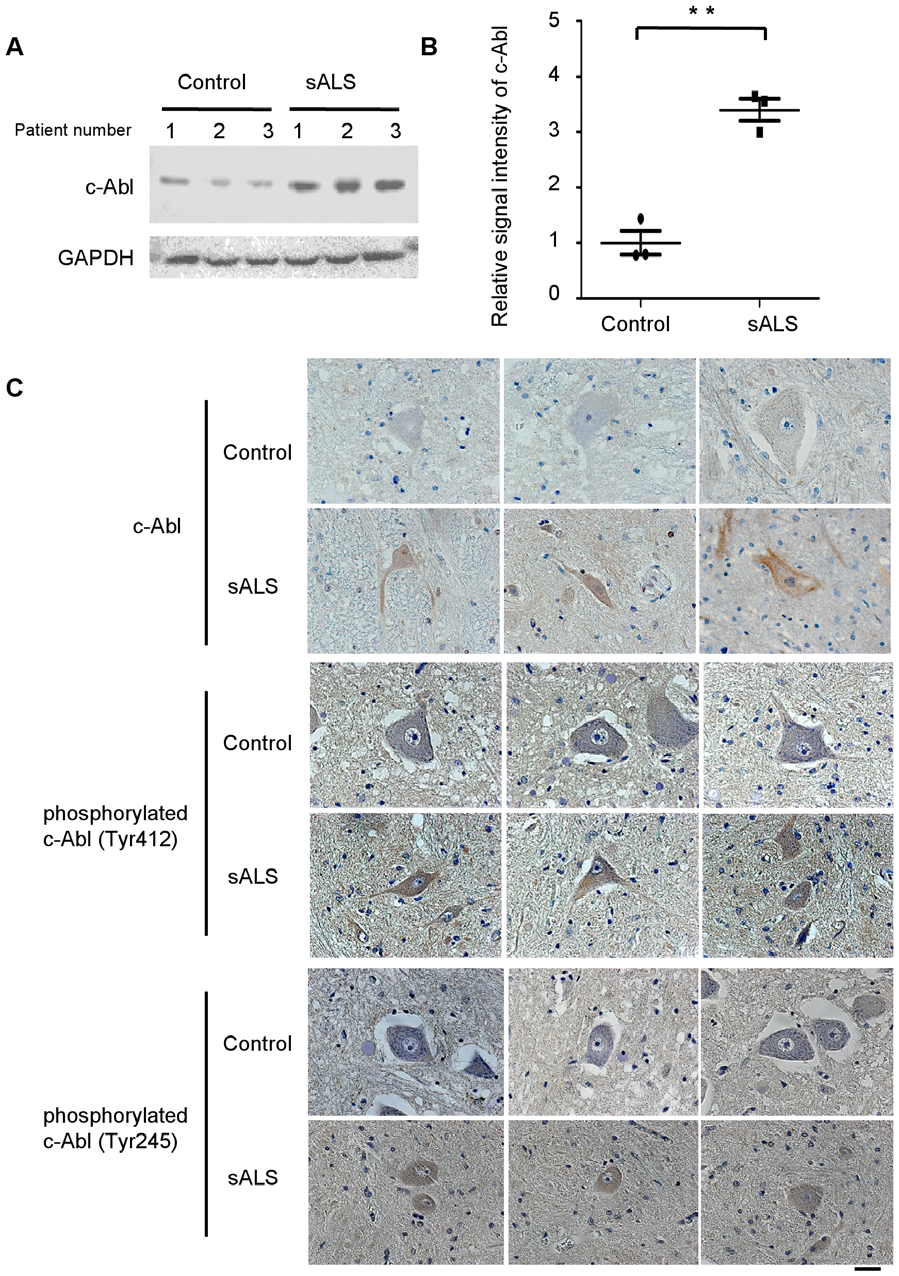
c-Abl upregulation and activation in affected motor neurons of sporadic ALS patients. A: The protein expression of total c-Abl was measured by western blot analysis using an anti-c-Abl antibody and the lumbar spinal cord tissue from sporadic ALS (sALS) cases and controls. GAPDH is shown as an internal control. The patient number refers to individual patients. B: Densitometric analysis using Image J software revealed a significant difference in the amount of total c-Abl protein in the lumbar spinal cords of sALS patients and controls (*P*<0.01). Data are presented as mean ± SEM. Statistical analysis was performed using Student's *t* test. **P<0.01. C: Immunohistochemical analysis using paraffin-embedded spinal cords from control and sALS patients was carried out by staining with anti-c-Abl, anti-phosphorylated c-Abl (Tyr412), and anti-phosphorylated c-Abl (Tyr245) antibodies. Scale bar: 50 µm.

To investigate the implications of c-Abl in human sALS, we next examined the expression and activation levels of c-Abl in post-mortem spinal cord specimens from sALS cases. Lumbar spinal cord tissue from 3 sALS cases and 3 control cases with no neurodegenerative disease were used for immunohistochemical and western blot analyses. Western blotting revealed a more than 3-fold increase in c-Abl protein in sALS (Fig. 8A, B). More intense c-Abl immunohistochemical signal was also observed in lumbar spinal cord sections from sALS cases compared to control cases (Fig. 8C). Immunoreactivity of phosphorylated c-Abl (Tyr245 and Tyr412) in motor neurons was also increased in sALS specimens compared to controls (Fig. 8C). These findings indicate that upregulation and activation of c-Abl in motor neurons occurs not only in G93A mice but also in sALS patients.

## Discussion

In this study, we established mouse motor neuronal cell lines in which either wild-type or mutant SOD1s were induced by doxycycline. We found that overexpression of mutant SOD1s induced expression and activation of c-Abl and decreased cell viability in a mouse motor neuron cell model. Furthermore, dasatinib, a BBB-permeable inhibitor of c-Abl, attenuated c-Abl phosphorylation and reduced the cytotoxicity induced by overexpression of mutant SOD1s. Dasatinib is a dual kinase inhibitor against c-Abl and c-Src family tyrosine kinases [31]. To clarify the specificity of c-Abl for the motor neuronal cytotoxicity, we performed cell proliferation and cell death assays with or without SU6656, which preferentially inhibits c-Src compared to c-Abl [32]. As shown in Fig. 3, dasatinib ameliorated the cytotoxic effects of mutant SOD1, whereas SU6656 did not. This finding indicates that c-Abl inhibition delays motor neuronal cell death caused by mutant SOD1. Our results are consistent with previous studies demonstrating that some apoptotic stimuli, such as amyloid beta and oxidative stress, also caused c-Abl activation [25], [29], and that imatinib, another c-Abl inhibitor, had an inhibitory effect on apoptotic pathways [28].

Our study also provides evidence that c-Abl upregulation and activation occur in the lumbar spinal cord of G93A mice. c-Abl activation has recently been reported to occur in animal models of Niemann-Pick type C and Alzheimer's disease [28], [33], but the present report is the first to demonstrate c-Abl activation in an animal model of ALS. Throughout the disease course of G93A mice, hyperphosphorylation and upregulation of c-Abl was apparent in the lumbar spinal cord. Notably, although apoptosis-related molecules such as c-Abl were expected to exert their function at a relatively late stage of disease [34], [35], the expression of c-Abl was increased at the presymptomatic stage. This unexpected result suggests that c-Abl may be an early player in the apoptotic cascade of ALS pathogenesis and thus a promising target to protect motor neurons against cytotoxic insults.

The currently available c-Abl inhibitors are imatinib, dasatinib, and nilotinib, all of which have been used for the treatment of CML, Ph+ALL, and gastrointestinal stromal tumor [36], [37], [38]. A number of studies have reported CNS relapse in patients treated with imatinib, which has poor BBB permeability [39], [40], [41], [42], [43], [44], while in contrast, Porkka et al. reported that dasatinib crossed the BBB and showed therapeutic efficacy against CNS CML tumors in a mouse model and in patients with CNS leukemia (Ph+ALL) [45]. The high BBB permeability of dasatinib is advantageous for the treatment of ALS, since it is expected to achieve a sufficient therapeutic concentration in the CNS. We demonstrated that dasatinib at a dose of 15 mg/(kg·day) or more delayed disease progression and extended the survival of G93A mice. Immunostaining of spinal cords clearly demonstrated a dose-dependent protective effect of dasatinib on motor neuron survival by inhibiting apoptosis. These results indicate that c-Abl plays an important role in the disease pathogenesis of ALS in G93A mice and is a promising therapeutic target for ALS.

Since the involvement of c-Abl upregulation and activation has been demonstrated in neuronal cell apoptosis [46], [47], we investigated whether upregulation of c-Abl is associated with an increased level of activated caspase-3, which correlates with apoptosis. Our results clearly showed that caspase 3 was activated in the spinal cords of G93A mice. Administration of dasatinib attenuated both c-Abl phosphorylation and caspase-3 activation in a dose-dependent manner. Thus, our results suggest that dasatinib ameliorates the phenotype of these animals by suppressing apoptotic cell death of motor neurons caused by mutant SOD1.

The examination of NMJs revealed that dasatinib successfully reversed the deinnervation of NMJs, an early pathological change reflecting motor neuron degeneration in mutant SOD1-mediated ALS [48]. Since levels of total and active c-Abl were increased in the spinal cords of G93A mice at the early stage of the disease (Fig. 4), dasatinib appears to improve NMJ function via c-Abl-mediated signaling. These findings suggest that dasatinib improved motor neuron function leading to amelioration of weight loss in G93A mice. They also demonstrate that the loss of synaptic contacts is a sensitive indicator of the beneficial effects exerted by dasatinib in G93A mice.

One possible explanation for the relatively small effects of dasatinib in this study is that the beneficial effects of this therapy on apoptosis were limited in motor neurons and could not reverse the physical dysfunction of the mice, despite the improvement in innervation at NMJs. Alternatively, dasatinib may not be capable of mitigating non-apoptotic pathways of motor neuron degeneration caused by mutant SOD1, since non-apoptotic programmed cell death has also been implicated in motor neuron damage in G93A mice [49]. Taken together, dasatinib may mitigate apoptotic events that occur at an early stage of the disease and partially improve motor neuron function via activation of c-Abl.

Using human postmortem spinal cord tissue, we demonstrated a significant increase in c-Abl expression in the spinal cord of sALS compared with non-ALS. Histochemical findings confirmed that c-Abl protein increased mainly in motor neurons. In addition, c-Abl phosphorylation was also increased in motor neurons in the affected area. These findings indicate that c-Abl abnormality is involved in human sALS cases as well as cellular and animal models of ALS. Thus far, not many drug candidates derived from research using mutant SOD1 transgenic animals have been successful in clinical trials for human sALS [50]. The implication of c-Abl in sALS as well as mutant SOD1-associated ALS supports the possible application of dasatinib as a candidate drug for sALS treatment. Our study showed that dasatinib treatment suppressed apoptosis and delayed disease progression in G93A mice, suggesting that dasatinib has a potential therapeutic value in humans, since apoptosis appears to be an important target of treatment development for ALS [35], [51].

In conclusion, the major findings of this study are (1) the observation of c-Abl upregulation and activation in the spinal cords of G93A mice at a relatively early stage of the disease; (2) the improved survival of G93A mice with concomitant suppression of c-Abl phosphorylation and caspase-3 activation upon administration of a BBB-permeable c-Abl inhibitor, dasatinib; and (3) increased c-Abl expression and phosphorylation in postmortem spinal cord tissues from sALS patients. Taken together, our results suggest that c-Abl is a novel therapeutic target for ALS.

## Materials and Methods

### Cell lines

The mouse motor neuron hybridoma line NSC-34 was provided by Dr. N.R. Cashman (University of Toronto; Toronto, Canada) [30]. Human wild-type and mutant (G93A and G85R) SOD1 cDNAs were subcloned from pcDNA3.1/SOD1 into lentiviral expression vectors (pLenti-CMV/TO, kind gifts from Dr. Eric Campeau at the University of Massachusetts Medical School) [52]. Lentiviral particles were produced in HEK293T cells (Open Biosystems, Huntsville, AL, USA) by transfection with Lipofectamine 2000 (Invitrogen, Eugene, OR, USA). Lentivirus-containing supernatant was collected 48 h after transfection and stored at −80°C. Details of the lentivirus system have been described previously [52]. We first transduced the Tet repressor into NSC-34 cells and selected a single clone (NSC-34-TetR14) that demonstrated good induction without leaky expression. NSC-34-TetR14 cells were stably transduced with lentivirus-Tet-on/SOD1, an inducible lentivirus expressing Myc-tagged wild-type or mutant SOD1.

### Cell culture

NSC-34 cells were grown in Dulbecco's modified Eagle's medium (DMEM) containing 10% fetal calf serum (FCS; Invitrogen). The tet-on inducible cell lines were grown in DMEM supplemented with 10% tetracycline-free FCS (Clontech, Mountain View, CA, USA). All cell lines used in this study were cultured at 37°C in an atmosphere of 5% CO_2_. We induced hSOD1 expression by adding 2 µg/ml doxycycline (Clontech) to the culture medium for the last 48 h of culture.

### Cell viability assay

Each of the cell lines (3,500 cells per well) were grown on collagen-coated 96-well plates with serum-free medium. MTS (3-(4,5-dimethylthiazol-2-yl)-5-(3-carboxymethoxyphenyl)-2-(4-sulfophenyl)-2H-tetrazolium)-based cell proliferation assays were performed after 48 h of induction with doxycycline (Dox, 2 µg/ml) using the CellTiter 96® AQueous One Solution Cell Proliferation Assay (Promega, Madison, WI, USA). Briefly, we added CellTiter 96® AQueous One Solution Reagent to each well of a 96-well assay plate containing the samples in culture medium. After incubation at 37°C for 1 h, absorbance at 490 nm was measured using a multiple-plate reader (Powerscan HT, Dainippon Pharmaceutical, Osaka, Japan), with assays carried out in triplicate.

### Cytotoxicity detection assay

Cell injury was quantitatively assessed by measurement of LDH released from damaged or destroyed cells into the extracellular fluid after 48-h induction of wild-type or mutant SOD1. The activity of LDH released into the culture medium was measured with a Cytotoxicity Detection kit (Roche Applied Science, Burgess Hill, UK) according to the manufacturer's protocol. Briefly, after 48 h of induction with doxycycline (Dox, 2 µg/ml), we added substrate mixture from the kit to each well of a 96-well assay plate containing the culture supernatant. Following incubation for 30 min, absorbance at 490 nm was measured using a multiple-plate reader (Powerscan HT, Dainippon Pharmaceutical, Osaka, Japan).

### Transgenic mice

Transgenic mice overexpressing the human SOD1 gene carrying the G93A mutation were purchased from the Jackson Laboratory (Bar Harbor, ME, USA) and maintained as hemizygotes by mating transgenic males with B6/SJLF1 females [53]. All animal experiments were performed in accordance with the National Institute of Health Guide for the Care and Use of Laboratory Animals and were approved by the Nagoya University Animal Experiment Committee.

### Chemicals

Dasatinib was provided by Bristol-Myers Squibb. Propylene glycol was purchased from Sigma Chemical Co. (St Louis, MO, USA). SU6656 was purchased from Calbiochem (Darmstadt, Germany). All other chemicals used were reagent grade or better.

### Drug formulation and administration

For oral administration, dasatinib was dissolved in a mixture of propylene glycol/water (50∶50). The administration volume was 0.01 ml/g. Ludolph et al. recommended that a total of 48 G93A mice should be used in a preclinical trial if 2 groups (treated animals and controls; n = 24 per group) are to be compared, and recommended that the number of animals should be increased for testing the dose-response effect of a drug [50]. Therefore, we allocated 28 mice to each treatment group for the survival analysis. From postnatal day 56, dasatinib was administered by oral gavage using a 5-days-on/2-days-off once-daily schedule (Monday through Friday) at doses of 5, 15, and 25 mg/(kg·day). Control mice received vehicle alone.

### Immunohistochemistry

Under pentobarbital anesthesia, mice were transcardially perfused with 20 ml phosphate buffer (pH 7.4). Tissues were postfixed overnight in 10% phosphate-buffered formalin and processed for paraffin embedding as previously described [54]. Transverse sections of spinal cord (6-µm thickness) were then deparaffinized with alcohol, rehydrated, and microwaved in 0.1 M citrate buffer (pH 6) as a pretreatment for antigen retrieval. Immunostaining was performed using the EnVision+ System-HRP (Dako, Glostrup, Denmark). Tissue sections were incubated with anti-c-Abl antibody (Abcam, Cambridge, MA) and anti-phospho-c-Abl (Tyr412 or Tyr245) antibody (Cell Signaling Technology, Beverly, MA, USA), both diluted 1∶100 in Dako antibody diluent (Dako) for immunohistochemical analysis. Counterstaining was performed using hematoxylin. For fluorescence microscopic analysis, after antigen retrieval, tissue sections were incubated with TNB-buffer (0.10 M Tris-HCl, 0.15 M NaCl, 0.5% BMP) for 30 min at room temperature to block non-specific antibody binding. Then spinal tissue sections were incubated with anti-phospho-c-Abl (Tyr412 or Tyr245) antibody (Cell Signaling Technology), both diluted 1∶100 in phosphate buffered saline (PBS) buffer, overnight at 4°C. After incubation with primary antibody, the sections were exposed to an appropriate secondary antibody conjugated to fluorescent dye and Topro-3 (Invitrogen) for 1 h at room temperature. Sections were visualized using a confocal microscope (LSM 710, Carl Zeiss, Oberkochen, Germany) under epifluorescent illumination. The intensity of immunostained neurons was semi-quantified using NIH Image J software (v 1.44, NIH, Bethesda, MD, USA).

### Assessment of motor function

The motor performance of mice was assessed weekly using an Economex Rotarod (Columbus Instruments, Columbus, OH, USA) starting at 42 days of age. Staying on the rod for more than 180 s was considered to be the normal performance level, as previously described [55].

### Western blot analyses

The spinal cords of dasatinib- and vehicle-treated mice were collected approximately 3 h after the final oral administration. Human and mouse spinal cords were snap frozen in liquid nitrogen, homogenized in ice-cold Cell Lytic-M Mammalian Cell Lysis/Extraction Reagent (Sigma), and centrifuged at 18,800× *g* for 15 min at 4°C. Protein concentration was determined by DC protein assay (Bio-Rad, Hercules, CA, USA). Western blotting was performed using standard procedures as described previously [56], [57]. Primary antibodies were used at the following concentrations: anti-SOD1, 1∶2,000 (Abcam); anti-Myc, 1∶1,000 (MBL, Nagoya, Japan); anti-tubulin, 1∶1,000 (Sigma); anti-c-Abl, 1∶1,000 (BD Transduction); anti-phospho-c-Abl (Tyr412), 1∶1,000 (Sigma); anti-phospho-c-Abl (Tyr245), 1∶1000 (Cell Signaling Technology); anti-glyceraldehyde-3-phosphate dehydrogenase (GAPDH), 1∶1,000 (Millipore, Billerica, MA); anti-phospho-c-Src (Tyr416), 1∶1,000 (Cell Signaling Technology); anti c-Src, 1∶1,000 (Cell Signaling Technology); and anti-cleaved caspase-3 (Asp175), 1∶1,000 (Cell Signaling Technology). Secondary antibody probing and detection were performed using the ECL Plus kit (GE Healthcare, Buckinghamshire, UK). For detection of phosphorylated c-Abl, antibody was diluted in Tris-buffered saline (TBS) with Tween (0.5%) containing 3% BSA, otherwise 5% fat-free milk in TBS with Tween (0.5%) was used as the antibody dilutant. Chemiluminescence signals were digitalized (LAS-3000 Imaging System; Fujifilm, Tokyo, Japan), and band intensities were quantified using Multi Gauge software version 3.0 (Fujifilm).

### Quantitative real-time PCR

Real-time PCR was performed as described previously [58]. In brief, total RNA from either mouse spinal cord or NSC-34 cells was reverse transcribed into first-strand cDNA using SuperScript II reverse transcriptase (Invitrogen). Real-time PCR was performed using QuantiTect SYBR Green PCR Master Mix and 0.4 M of each primer (Qiagen, Valencia, CA, USA), and the product was detected using the CFX96™ real-time system (Bio-Rad Laboratories). The reaction conditions were 95°C for 15 min, followed by 40 cycles of 15 s at 94°C, 30 s at 55°C, and 30 s at 72°C. The expression level of GAPDH was quantified and used as an internal standard control. The primers used were 5′-TCGTTACCTCCAAAGGCTGCTC-3′ and 5′-ATGGCGGTGTCTGGCTATTCA-3′ for c-Abl and 5′-TCAAGAAGGTGGTGAAGCAG-3′ and 5′-GTTGAAGTCGCAGGAGACAA-3′ for GAPDH.

### Motor neuron assessment by immunohistochemical analysis

At age 120 days, 8 animals from each treatment group were sacrificed, and the lumbar spinal cords (L1-3) were collected. The samples were embedded in paraffin, and 6-µm sections were prepared. Spinal cord tissue sections were immunostained with anti-ChAT antibody (Millipore) diluted 1∶1,000 in Dako antibody diluent (Dako) using the EnVision+ System-HRP (Dako). ChAT-immunoreactive neurons in the ventral horn of the lumbar spinal cord (L1–3) were counted in 3 sections taken at 60-µm intervals, and the mean total number of ChAT-immunoreactive neurons was compared between treatment groups. The area (pixels) of ChAT-immunoreactive neurons was analyzed using NIH Image J software (NIH). ChAT-positive cells with an area greater than 100 µm^2^ were presumed to be motor neurons.

### NMJ assessment by immunohistochemical analysis

At the age of 120 days, 8 animals from each treatment group were sacrificed, and quadriceps femoris specimens were quickly frozen in liquid nitrogen. The samples were mounted in Tissue-Tek OCT compound (Sakura, Tokyo, Japan), and 30-µm cryostat sections were prepared from the frozen tissues. Frozen sections were fixed in acetone for 5 min and then incubated with TNB-buffer (0.10 M Tris-HCl, 0.15 M NaCl, 0.5% BMP) for 15 min at room temperature to block non-specific antibody binding. Sections were incubated with primary antibodies and alpha-BuTX overnight at 4°C. The following primary antibodies were used: anti-synaptophysin diluted 1∶100 (Cell Signaling Technology) and anti-SMI31, 1∶100 (COVANCE, Princeton, NJ, USA). Alpha-BuTX biotin-XX-conjugate diluted 1∶80 was purchased from Molecular Probes (Eugene, OR, USA). After washing with PBS, the sections were exposed to appropriate secondary antibody and streptavidin-conjugated fluorescent dye for 1 h at room temperature, then washed with PBS again and mounted. Sections were examined and photographed using a confocal microscope (LSM 710, Carl Zeiss) under epifluorescent illumination.

### Human sporadic ALS samples

Spinal cord specimens were obtained at autopsy from 3 pathologically confirmed cases of sALS (2 men, aged 71 and 73 y, and 1 woman, aged 53 y) and 3 cases of non-neurodegenerative disease. Lumbar spinal cord tissue was either homogenized for western blot analysis or embedded in paraffin for immunohistochemical analysis. The collection of autopsied human tissues and their use for this study were approved by the Ethics Committee of Nagoya University Graduate School of Medicine, and written informed consent was obtained from the patients' next-of-kin. Experimental procedures involving human subjects were conducted in conformance with the principles expressed in the Declaration of Helsinki.

### Statistical analyses

Statistical analyses were performed using Prism software (GraphPad Software, La Jolla, CA, USA). Biochemical data were statistically analyzed using Student's t test or 1-factor factorial ANOVA followed by appropriate post-hoc tests. Survival data was analyzed by log-rank tests, and body weight change was analyzed by 2-factor factorial ANOVA. *P* values of 0.05 or less were considered to be statistically significant.

## Supporting Information

Figure S1 **Increased phosphorylated c-Abl in spinal cords of G93A mice.** A: The distribution of phosphorylated c-Abl proteins was analyzed by immunohistochemical staining of paraffin-embedded spinal cord sections from G93A mice (10, 14, and 18 weeks old) and control littermates (20 weeks old) using antibodies directed against phosphorylated c-Abl (Tyr245 and Tyr412). The spinal sections were immunostained with anti-ChAT (red) and anti-phosphorylated c-Abl (Tyr245 or Tyr412) (green) antibodies together with Topro-3 (blue). Representative immunostained motor neurons visualized with confocal laser scanning microscopy are shown. Scale bar: 50 µm. B: The intensity of motor neurons labeled with anti-phosphorylated c-Abl (Tyr245) and anti-phosphorylated c-Abl (Tyr412) antibodies shown in A was quantified (n = 3 mice per group). Phosphorylated c-Abl immunoreactivity with both antibodies was significantly increased in the spinal cords of G93A mice (*P*<0.01). The value was standardized to that of the fluorescence intensity of control mice. Statistics were evaluated using 1-way ANOVA with Dunnett's post-hoc test. **P<0.01.
(TIF)

Figure S2 **Dasatinib reduced c-Abl phosphorylation (Tyr412) in G93A mice.** A: Phosphorylated c-Abl (Tyr412) protein was analyzed by immunohistochemical staining of paraffin-embedded spinal cord sections from dasatinib-treated G93A mice (0, 5, 15, and 25 mg/(kg·day)) using an antibody against phosphorylated c-Abl (Tyr412). The spinal sections were fluorescently immunostained with anti-ChAT (red) and anti-phosphorylated c-Abl (Tyr412) (green) antibodies together with Topro-3 (blue). Representative immunostained motor neurons visualized with confocal laser scanning microscopy are shown. Scale bar: 50 µm. B: The intensity of the cells stained with anti-phosphorylated c-Abl (Tyr412) was quantified. The mice were administered the indicated amounts of dasatinib daily from postnatal day 56 to day 120 (n = 3 mice per group). Immunoreactivity against phosphorylated c-Abl (Tyr412) was significantly decreased in dasatinib-treated G93A mice (15 mg/(kg·day) or more) compared to vehicle-treated G93A mice (*P*<0.01, 15 mg/(kg·day) and 25 mg/(kg·day)). The value was standardized to that of the fluorescence intensity of vehicle-treated G93A mice. Statistics were evaluated using 1-way ANOVA with Dunnett's post-hoc test. **P<0.01.
(TIF)
