# Supplementary figures and images for: c-Abl Inhibition Delays Motor Neuron Degeneration in the G93A Mouse, an Animal Model of Amyotrophic Lateral Sclerosis
Source: PLoS One. 2012 Sep 25;7(9):e46185. doi: 10.1371/journal.pone.0046185 (PMC3458026; doi:10.1371/journal.pone.0046185)

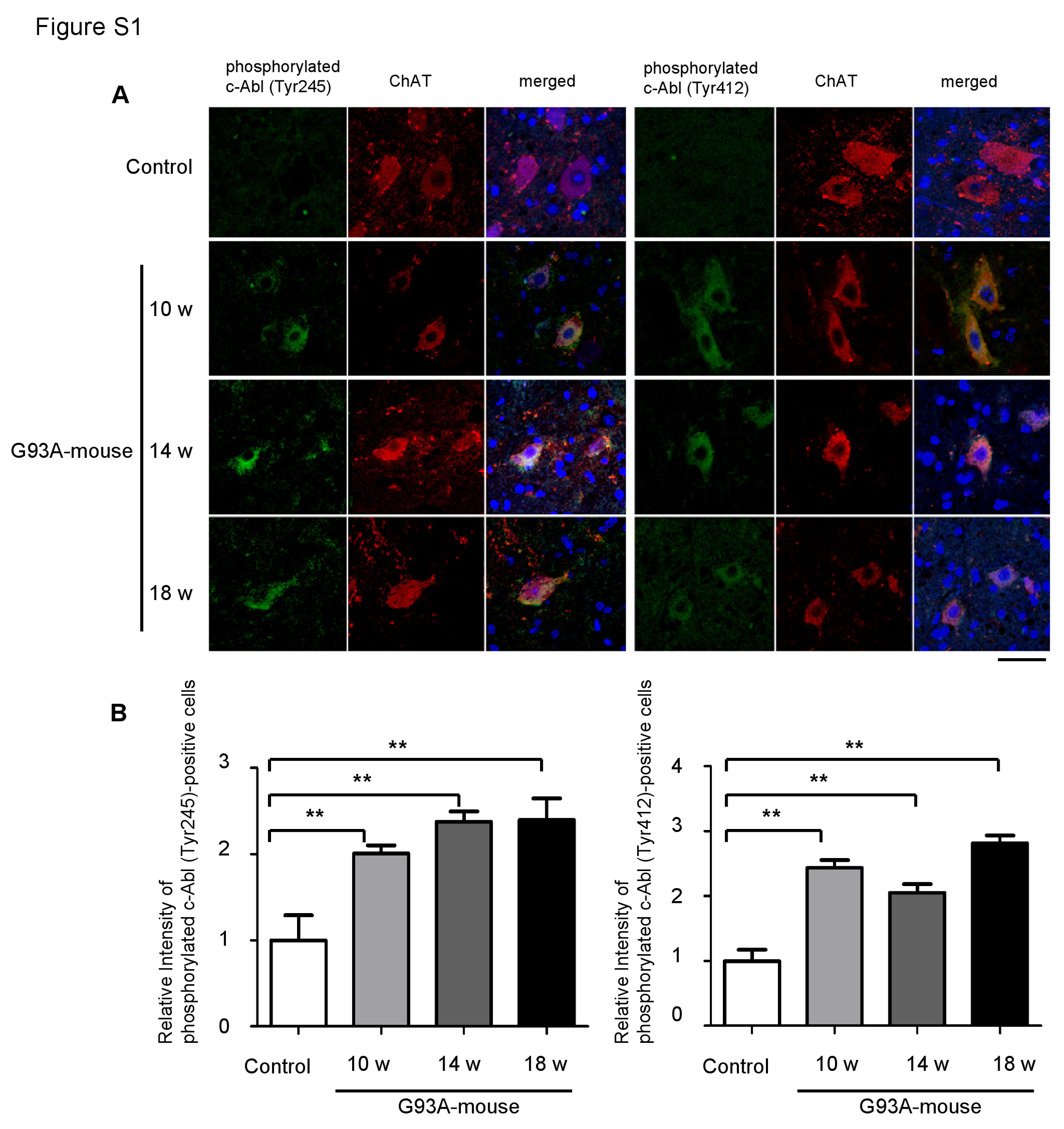

Supplement: Figure S1 — Increased phosphorylated c-Abl in spinal cords of G93A mice. A: The distribution of phosphorylated c-Abl proteins was analyzed by immunohistochemical staining of paraffin-embedded spinal cord sections from G93A mice (10, 14, and 18 weeks old) and control littermates (20 weeks old) using antibodies directed against phosphorylated c-Abl (Tyr245 and Tyr412). The spinal sections were immunostained with anti-ChAT (red) and anti-phosphorylated c-Abl (Tyr245 or Tyr412) (green) antibodies together with Topro-3 (blue). Representative immunostained motor neurons visualized with confocal laser scanning microscopy are shown. Scale bar: 50 µm. B: The intensity of motor neurons labeled with anti-phosphorylated c-Abl (Tyr245) and anti-phosphorylated c-Abl (Tyr412) antibodies shown in A was quantified (n = 3 mice per group). Phosphorylated c-Abl immunoreactivity with both antibodies was significantly increased in the spinal cords of G93A mice (P<0.01). The value was standardized to that of the fluorescence intensity of control mice. Statistics were evaluated using 1-way ANOVA with Dunnett's post-hoc test. **P<0.01. (TIF) [file pone.0046185.s001.tif]

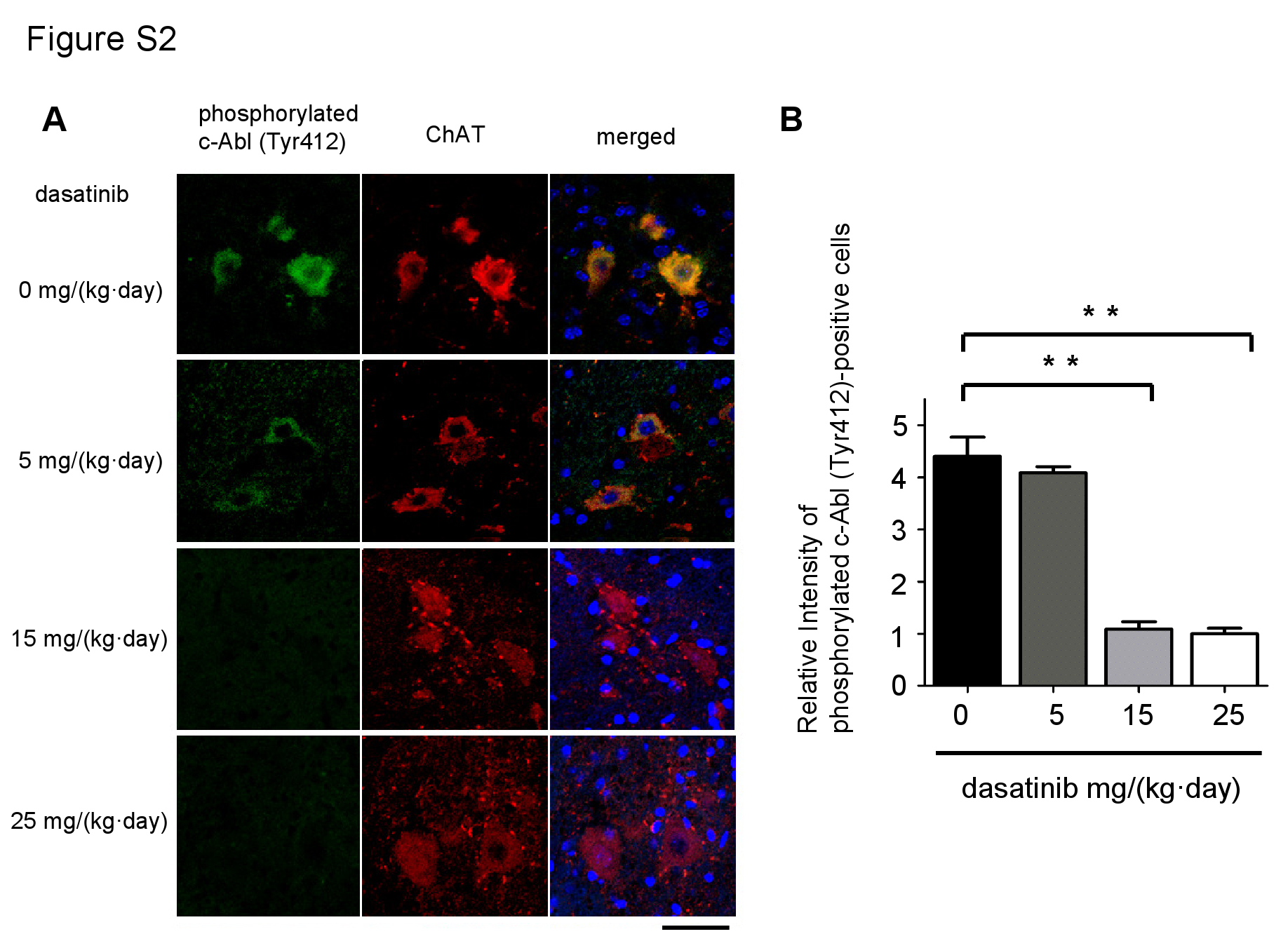

Supplement: Figure S2 — Dasatinib reduced c-Abl phosphorylation (Tyr412) in G93A mice. A: Phosphorylated c-Abl (Tyr412) protein was analyzed by immunohistochemical staining of paraffin-embedded spinal cord sections from dasatinib-treated G93A mice (0, 5, 15, and 25 mg/(kg·day)) using an antibody against phosphorylated c-Abl (Tyr412). The spinal sections were fluorescently immunostained with anti-ChAT (red) and anti-phosphorylated c-Abl (Tyr412) (green) antibodies together with Topro-3 (blue). Representative immunostained motor neurons visualized with confocal laser scanning microscopy are shown. Scale bar: 50 µm. B: The intensity of the cells stained with anti-phosphorylated c-Abl (Tyr412) was quantified. The mice were administered the indicated amounts of dasatinib daily from postnatal day 56 to day 120 (n = 3 mice per group). Immunoreactivity against phosphorylated c-Abl (Tyr412) was significantly decreased in dasatinib-treated G93A mice (15 mg/(kg·day) or more) compared to vehicle-treated G93A mice (P<0.01, 15 mg/(kg·day) and 25 mg/(kg·day)). The value was standardized to that of the fluorescence intensity of vehicle-treated G93A mice. Statistics were evaluated using 1-way ANOVA with Dunnett's post-hoc test. **P<0.01. (TIF) [file pone.0046185.s002.tif]
